# Physiological evaluation of the emotional regulation of patients with hereditary angioedema

**DOI:** 10.1186/s13030-025-00348-6

**Published:** 2026-01-06

**Authors:** Laurent Sparrow, Christelle Duprez, Louise Richez, Michel Raguet, Louis Terriou, Isabelle Citerne, Sébastien Sanges, Launay David

**Affiliations:** 1 Univ. Lille, CNRS, UMR 9193 - SCALab - Sciences Cognitives et Sciences Affectives, Lille, F 59000 France; 2 AMSAO - Association des Malades Souffrant d’Angio edèmes, 31 Rue du Chateaufort, Orsay, 91400 France; 3 CHU Lille, Département de Médecine Interne et Immunologie Clinique, Lille, F-59000 France; 4 Centre de Référence des Angioedèmes à Kinines (CREAK), Lille, F- 59000 France; 5 Univ. Lille, U1286 - INFINITE Institute for Translational Research in Inflammation, Lille, F-59000 France; 6 Inserm, Lille, F-59000 France

**Keywords:** Hereditary angioedema, Electrodermal activity, Heart rate variability, Stress, Kallikrein

## Abstract

**Objective:**

Hereditary angioedema (HAE) is a rare disease that presents with recurrent attacks that can be triggered by stress or other emotions. The emotional impact of HAE is well-documented, although mainly via self-reported questionnaires. In this study, we used physiological measures to investigate stress regulation in patients with HAE and compared them to patients with immune thrombocytopenia (ITP), a disease that also progresses with attacks but without emotional triggers.

**Methods:**

Twenty-six adult patients with HAE or ITP (13 each) were included in the study. Patients were asked to perform several computerized tasks that activated different branches of the autonomic nervous system (ANS). Heart rate variability and electrodermal activity were measured during the experimental tasks and a medical consultation that followed the experiments.

**Results:**

Patients with HAE did not differ from patients with ITP in their physiological responses. In the reactivity tasks, a non-significant correlation significance was observed between reaction times (RTs) and peak spread in the HAE and ITP groups only for male patients and in a different way for HAE (negative correlation) and ITP (positive correlation). During medical consultations, an association between the physician’s perception of the patient’s stress and the patient’s physiological measures of stress was found only in female HAE patients, but without significance.

**Conclusions:**

The ANS of patients with HAE and ITP was similarly and abnormally activated, with a possible effect by sex. These results shed new light on the role of emotional regulation in chronic diseases.

**Trial registration:**

This article does not report the results of a health care intervention on human participants. The research was not preregistered in any registry. The analysis plan was not preregistered in an independent, institutional registry. Data, analytic methods, and study materials are not made directly available.

**Supplementary Information:**

The online version contains supplementary material available at 10.1186/s13030-025-00348-6.

## Introduction

Hereditary angioedema (HAE) is a rare genetic disorder characterized by recurrent attacks of non-pruriginous edema that persists for several days on specific body parts. HAE presents in various forms, with types I and II resulting from C1-esterase inhibitor (C1-INH) deficiency or dysfunction, primarily due to mutations in the *SERPING1* gene [1]. This mutation disrupts the inhibitory function of C1-INH, causing bradykinin overproduction, increased vascular permeability, and edema [2–5]. The estimated prevalence of HAE is around 1:40,000, with estimates ranging from 1:10,000 to 1:50,000 [6]. The natural course of HAE comprises recurrent attacks, which are self-resolving, separated by remission periods. Attacks may affect the face, respiratory tract, body extremities, and abdomen. The involvement of the airway, in 25% of cases, can result in fatal asphyxiation if the attack is not promptly managed [7]. While attacks can occur spontaneously, a variety of triggers, including positive and negative emotions, particularly emotional stress, is commonly reported by patients [8, 9]. Attack severity and frequency vary among individuals, ranging from once a year to three times a week [1].

Pain resulting from swelling, characterized by subcutaneous tension or burning, often decreases engagement in daily activities, significantly affecting the quality of life [2, 4]. Abdominal attacks, characterized by abdominal pain, nausea, vomiting, or diarrhea, also have detrimental effects on quality of life. In addition, patients may experience a fear of death, particularly in response to swollen airways that hinder normal breathing. This fear, along with the anticipation of attacks and edema-induced pain, induces anxiety in patients, which further diminishes their quality of life [1, 2]. Suboptimal management of the disease may also exacerbate these issues. These aspects highlight the substantial physical and psychological effects of HAE on patients, including an increased risk of anxiety and depression [10–12].

HAE presents diagnostic challenges due to its rarity, leading to prolonged diagnostic delays and, in some cases, unnecessary procedures like surgical intervention for abdominal attacks [13, 14]. Although advances in medical treatment have substantially improved patient care, with prophylaxis and individualized drug therapies for acute attacks now available [15], conventional therapies such as danazol and tranexamic acid often come with significant side effects that affect patient well-being [16, 17].

Recent developments in the treatment of patients with HAE include kallikrein inhibitors such as lanadelumab and berotralstat, which have shown remarkable efficacy in reducing attack frequency and improving patient quality of life [18]. Lanadelumab, available since 2018 and administered via injection, has demonstrated up to a 90% reduction in mean attack rate [19, 20]. While lanadelumab may induce adverse effects, it typically has a minimal impact on quality of life. Berotralstat, available since 2021 and in oral form, also reduces attack rates and improves quality of life, with manageable digestive side effects [17, 21]. As reported in literature reviews by Levy et al. [22] and Chong-Neto [18], the use of kallikrein inhibitors significantly improves quality of life in all domains, including emotional domains, with patients receiving these treatments showing better quality of life than patients using on-demand treatment only. As the number of attacks decreases and they experience attack-free periods, patients may feel less anxious about potential attacks, and the daily-life burden in functioning may be alleviated [23].

Stress is a multifaceted state in which physiological, endocrinological, and psychological responses triggered by events that disrupt homeostasis are involved [24]. These events activate the sympathetic branch of the autonomic nervous system (ANS) (the “fight or flight” reaction), mobilizing the body’s resources for emergencies [25, 26]. In contrast, the parasympathetic branch promotes resource replenishment (the “rest and digest” reaction). Dysregulation of these branches, characterized by sympathetic hyper-reactivity and parasympathetic hypo-reactivity, should be transient and tied to stress stimuli, with the body returning to a resting state after the trigger [27, 28].

There are different ways to measure emotional activation (e.g., behavioral, subjective, and physiological). The activity of the two branches of the ANS can be measured with cardiac indicators such as heart rate variability (HRV) and electrodermal indicators such as electrical conductance of the skin corresponding to electrodermal activity (EDA). HRV is a measure of the parasympathetic branch and refers to continuous fluctuations in the duration between consecutive cardiac contractions (R-R intervals in milliseconds - ms; i.e., a measure of the inter-beat distance between two cardiac QRST complexes) over a given period. HRV is a simple indicator of changes in the level of activity of the ANS during stressful situations or states. HRV analysis involves measuring R-R intervals. Frequency analysis over a frequency band ranging from 0.15 Hz to 0.4 Hz enables high frequencies (HFs) to be analyzed. These frequencies are linked to the efferent branch of the vagus nerve (under parasympathetic influence). As breathing increases, this frequency band also increases. HF measurement is a way of highlighting parasympathetic activity (see the meta-analysis by Castaldo et al. [29]). HRV has been related with sex, with HRV being higher at rest in women than in men [30–33]. A stronger correlation between resting HRV and access to emotional regulation strategies has been reported for women than for men. Additionally, women have been reported to experience greater difficulties regulating their emotions when resting HRV levels are lower. This suggests that the lower resting HRV levels in women may have more significant implications for their emotional regulation than for men [32, 33]. However, HF measurements appear similar for men and women [31].

The ANS of patients with HAE may be altered, with an over-activation of the sympathetic branch and an under-activation of the parasympathetic branch during the resting state compared with a control population. Wu et al. [34] compared the physiological and biological indicators of stress (electrocardiogram, beat-to-beat blood pressure, and respiratory activity) of 23 adult HAE patients (20 HAE type 1, 3 HAE type 2; 6 male, mean age 47.5+/-11.4 years) with those of 24 healthy volunteers (eight male, mean age 45.3 +/-10.6 years). Participants were placed in a task situation that mobilized the orthostatic axis and involved the different branches of the ANS by being placed on a tilting table, inducing a physiological stress. Measurements were taken at rest and when the body was inclined at 75° degrees. Sympathetic branch activity was measured by HRV indicators: Low frequencies (LFnu, an indicator of sympathetic modulation), HF, and the LF/HF ratio, an index of sympathovagal balance. The results showed that in the tilted position (activation of the sympathetic axis), HF was higher in the patients with HAE than in controls, with lower variability. Furthermore, although LFnu significantly increased after this task in both groups, the LF/HF ratio significantly increased only in controls (Wu et al. [34]).

Wu et al. [34] observed a distinct physiological response pattern in patients with HAE that was characterized by sympathetic hyperactivity and parasympathetic hypoactivity during a postural task, but to better understand sympathetic activation it is crucial to consider electrodermal measurements. EDA effectively assesses sympathetic branch activity and is a reliable neurovegetative indicator of cognitive states [35]. EDA reflects electrical variations in the skin associated with sweat gland function, with two components: skin conductance level (SCL) and skin conductance response (SCR) [35, 36]. SCL represents the tonic activity of the sympathetic system, while SCR indicates phasic variations in EDA, reflecting emotional activation. The frequency of SCR peaks per period measures emotional reactivity, with increased frequency indicating heightened arousal and decreased sensory thresholds [37, 38]. Therefore, examining EDA can provide valuable insights into sympathetic activation and the emotional responses of patients with HAE.

The study by Wu et al. [34] focused solely on physical, stress-inducing tasks, while autonomic deregulations in psychiatric patients have been explored using tasks involving psychological stressors. For instance, Hu et al. [39] used heart rate variability indicators to demonstrate sympathetic hyperactivity and parasympathetic hypoactivity in anxious patients during medical consultations. Tasks involving psychological stressors are relevant when investigating the physiological aspects of the emotions of patients with HAE, given that stress and emotions can trigger disease attacks. Furthermore, HRV measurements have been linked to emotional regulation, with higher resting HRV associated with better adaptation to stressors and cognitive responses to emotional stimuli [40]. Given these findings, it is crucial to investigate the emotional regulation of patients with HAE with physiological indicators and to examine autonomic regulation in psychologically stress-inducing situations.

### Objectives

The objectives of this study were to determine whether patients with HAE exhibit specific patterns of emotional activation in terms of their physiological responses and to assess whether they show more dysregulations in the balance between the two branches of the ANS than control patients with another rare disease that also involves acute attacks which are not triggered by emotional stress (immune thrombocytopenia, ITP). Indeed, we hypothesized that the emotional dysregulation associated with HAE, if confirmed, could be a “prophylactic” mechanism aiming at preventing further attacks. As such, ITP, an acquired autoimmune disorder characterized by acute drops in platelet count that potentially result in bleeding and that occurs independently of the patient emotional state would be an adequate control for the purpose of this study. Acute ITP attacks are mostly managed by corticosteroids and/or intravenous immunoglobulins.

We used an experimental task known to activate the two branches of the ANS, with HRV and EDA measurements. We also sought to evaluate whether a physician ‘s perception of patient stress during a follow-up medical consultation correlated with the patient’s physiological activation pattern, an objective indicator of patient stress.

## Methods

### Participants

The study included patients with HAE-C1 INH type I or II and patients with ITP. All were over 18 years old and provided informed consent to participate in the study. Typically, patients with ITP share a similar age profile with HAE patients. Furthermore, both diseases involve attacks separated by asymptomatic periods. Patients with ITP are also regularly monitored by medical appointments, justifying the choice of this population as a control group.

### Procedure

The study was approved by the ethics committee of the University of Lille (Comité d’Ethique et de Recherche – CER-Lille) in January 2020 (reference number: 2019-393-S78). Patients were recruited from the Internal Medicine Department of Lille University Hospital from January 2022 to May 2023. They were informed of the study prior to a follow-up medical consultation in the Department, and an information letter was sent to them by email. If they were interested in participating in the study, they were asked to arrive early on the day of their consultation. A research assistant ensured that the patient had read and understood the information letter and collected the patient’s written informed consent. Patients were then led to a dedicated room in the Department where they were fitted with an Empatica E4 wristband, a wireless device designed for real-time, continuous HRV and EDA data acquisition. The device, worn like a watch, is internationally recognized and used in research and healthcare to record physiological data [41].

To obtain physiological measurements at rest (baseline measurements, for at least 2 min), the patients were asked to complete a sociodemographic data sheet while wearing the wristband. Each patient also completed a medical data sheet, including the number of attacks, attack locations, and treatments received.

The patients were then asked to perform three tasks on a laptop dedicated to the study: (1) a reactivity task (i.e., pressing a key as soon as a stimulus appears on the screen) to measure sympathetic activity; (2) a breathing task that activates the parasympathetic system (short inspiration and long expiration breathing exercise [42; 3], a cognitive task designed to induce cognitive stress (a mathematical operation was displayed on the screen and the participant gave the result in writing) (see supplementary material).

The entire experiment took less than 30 min. Afterward, patients went to their medical follow-up consultation in the ward. At the start of the consultation, a research assistant activated the wristband to start it recording, then stopped it once the consultation was over. The research assistant did not assist the medical encounter. At the end of the consultation, the doctor was asked to indicate how stressed he/she perceived the patient to have been during the consultation: on a scale from 0 = not at all to 10 = completely stressed. The research assistant then collected the bracelet and answeredany question the patient might have.

### Statistical analyses

Various indicators were used. The number of peaks per period is a measure of phasic EDA, reflecting emotional activation. When stress mechanisms are activated, central alert systems increase while perceptual thresholds decrease, leading to a higher frequency of peaks without changes in amplitude. The peak spread (area under the curve) reflects the temporal width of a phasic electrodermal response. This indicator indexes the strength of the sympathetic nervous system’s phasic activation in response to a stimulus (typically emotional, attentional, or cognitive). In addition, the temporal extent of the peak can be interpreted as an index of the persistence or duration of sympathetic activation [36]. Heart rate variability (HRV) can be divided into frequency bands. High-frequency HRV (HF-HRV) reflects vagal activity and is considered a reliable index of parasympathetic function [43].

Indicators measured during each phase of the experiment were compared for the HAE and ITP patient groups. Indicators were the number of peaks per period (phasic EDA) in the reactivity task, HF-HRV (ms2) in the breathing task, and number of peaks per period (phasic EDA) and HF-HRV (ms2) in the cognitive load task (Supplementary material 1). For the reactivity phase, a linear correlation with Pearson’s r was calculated for the reaction times and peak spread in each patient group. For HAE, treatment received (kallikrein inhibitor or other) and effects by sex were also assessed.

For the consultation phase, the number of peaks per period was also compared. Linear correlations between physiological stress (phasic EDA) and physician-perceived stress (self-reported measures from 0 to 10) were performed. Percent increase in HF-HRV was measured for the breathing and reactivity tasks with reference to a baseline level for between group comparison.

Comparisons of the patient groups were made using Student’s t-test for each hypothesis, where the normality of the data was verified using the Shapiro-Wilk test and a test of homogeneity of variances (Levene’s test). When the data was not normal, a Mann-Whitney U test was performed. All tests were two-tailed with a significance level of *p* < 0.05.

## Results

### Patient characteristics

A total of 13 patients with HAE (*n* = 7 female, *Mean age =* 46.6, *SD* = 14.6) and 13 with ITP (*n* = 7 female, *Mean age* = 45.23, *SD* = 21.26) were included in the study. The two patient groups were comparable in terms of age (*t* (24) =-0.17, *p* = 0.87) and male/female distribution (Chi-squared = 0.00; *p* = 1). All patients with HAE were type 1, and 83.3% of those with ITP had an isolated form of the disease (Table 1). Medical and sociodemographic characteristics of the participants are shown in Table 1.

**Table 1:** Demographics and clinical data

	HAE		ITP	
	Female	Male	Female	Male
*n* (%)	7 (53.85%)	6 (46.15%)	7 (53.85%)	6 (46.15%)
Age *(M*,* SD)*	55.86 (12.62)	35.83 (7.93)	34.86 (12.05)	57.33 (24.19)
**Professional activity (*n***, **%)**				
Craftsmen, shopkeepers and company directors	0	1 (16.66%)	0	1 (16.66%)
Managers and higher intellectual professions	1 (14.28%)	1 (16.66%)	1 (14.28%)	2 (33.33%)
Intermediate professions	0	0	1 (14.28%)	0
Employees	2 (28.57%)	3 (50%)	2 (28.57%)	1 (16.66%)
Manual workers	0	0	1 (14.28%)	0
Non-active	1 (14.28%)	1 (16.66%)	1 (14.28%)	0
Student	0	0	1 (14.28%)	0
Retired	3 (42.86%)	0	0	2 (33.33%)
**Education (*n***, **%)**				
No diploma	1 (14.28%)	1 (16.66%)	1 (14.28%)	0
Lower secondary education	3 (42.86%)	3 (50%)	0	1(16.66%)
High school – upper secondary education	0	0	3 (42.86%)	1(16.66%)
Master degree	2 (28.57%)	1 (16.66%)	2 (28.57%)	2 (33.33%)
Bachelor degree	0	1 (16.66%)	1 (14.28%)	2 (33.33%)
**Marital status (*n***, **%)**				
Single	2 (28.57%)	1 (16.66%)	4 (57.14%)	2 (33.33%)
In a couple	5 (71.43%)	5 (83.33%)	3 (42.86%)	4 (66.66%)
**Age at diagnosis**^**a**^ **(***M*,* SD)*				
	19.6 (9.71)	18.8 (15.41)^a^	28 (6.66)	46.6 (28.72)
**Type of disease**				
**HAE (*n***, **%)**				
Type 1	7 (100%)	6 (100%)	-	-
Type 2	0	0	-	-
**IT (*n***, **%)**				
Isolated immunological thrombocytopenia	-	-	5 (71.43%)	5 (83.33%)
Immune thrombocytopenia associated with another disease	-	-	2 (28.57%)	1 (16.66%)
**Clinical indicators at diagnosis**				
**HAE**				
C1 inhibitor (mg/L) (*M*,* SD*)	80,62 (45,42)		-	
C4 assay (mg/L) (*M*,* SD*)	73,85 (50,29)		-	
**IT**				
Blood platelet level (*M*,* SD*)			19166,67 (16677.87)	
Khellaf score (*M*,* SD*)			2.5 (2.43)	
**Sites previously involved in angioedema attacks: HAE (*n***, **%)**				
Otorhinolaryngeal with laryngeal involvement	5 (71.43%)	1 (16.66%)		
Otorhinolaryngeal without laryngeal involvement	1 (14.28%)	2 (33.33%)		
Face	6 (85.71%)	3 (50%)		
Abdomen	6 (85.71%)	6 (100%)		
Upper limbs	7 (100%)	5 (83.33%)		
Lower limbs	7 (100%)	5 (83.33%)		
External Genitals	5 (71.43%)	2 (33.33%)		
**Sites previously involved in bleeding: ITP (*n***, **%)**				
Skin (purpura, ecchymosis)	-	-	7 (100%)	6 (100%)
Nasal mucosa (epistaxis)	-	-	0	0
Oral mucosa (hemorrhagic bullae)	-	-	0	2 (33.33%)
Digestive tract (hematemesis, rectal discharge, melena)	-	-	0	0
Urinary tract (macroscopic hematuria)	-	-	0	0
Genital tract (menorrhagia, metrorrhagia)	-	-	0	0
Central nervous system			0	0
**Number of attacks (HAE) / relapses ( ITP) over the last six months** ^b^(*M*,* SD*)	5.83 (6.01) ^b^	4.60 (4.72) ^b^	0.57 (1.13)	0.50 (0.55)
**Presence of a severe attack/relapse in the last six months** (*n*, %)	5 (71.43%)	3 (50%)	3	3
**Attack triggers (for HAE) / relapse triggers (for ITP) (*n***, **%)**				
Stress	5 (71.43%)	6 (100%)	0	0
Physical trauma	4 (57.14%)	3 (50%)	0	0
Physical activity	4 (57.14%)	4 (66.66%)	0	0
Drugs	1 (14.28%)	1 (16.66%)	0	0
Surgery	2 (28.57%)	1 (16.66%)	0	0
Environmental factors	1 (14.28%)	3 (50%)	0	0
Infection	2 (28.57%)	2 (33.33%)	2 (28.57%)	5 (83.33%)
Others (pregnancy)	1 (14.28%)	0	0	0
**Attack treatment (*n***, **%)**				
**HAE**				
Icatibant	7 (100%)	5 (83.33%)	-	-
Tranexamic acid	7 (100%)	6 (100%)	-	-
C1 inhibitor	0	1 (16.66%)	-	-
None	1 (14.28%)	1 (16.66%)	-	-
**ITP**				
Corticosteroids	-	-	6 (85.71%)	6 (100%)
intravenous immunoglobulins	-	-	5 (71.43%)	5 (83.33%)
Thrombopoietin agonists	-	-	1 (14.28%)	3 (50%)
None				
**Long-term treatment (*n***, **%)**				
**HAE**				
Danazol	0	2 (33.33%)	-	-
Tranexamic acid	0	0	-	-
C1 inhibitor	1 (14.28%)	1 (16.66%)	-	-
Lanadelumab	5 (71.43%)	3 (50%)	-	-
Berotralstat	1 (14.28%)	0	-	-
**ITP**				
Danazol	-	-	0	1 (16.66%)
Disulone	-	-	1 (14.28%)	1 (16.66%)
Thrombopoietin agonists	-	-	2 (28.57%)	4 (66.67%)
Rituximab	-	-	0	2 (33.33%)
Splenectomy	-	-	0	0
Other	-	-	1 (14.28%)	0

Abbreviations: N, number of subjects; M, mean; SD, standard deviation; HAE, hereditary angioedema; IT, immunological thrombocytopenia

^a^ age at diagnosis was missing for one male HAE patient

^b^ number of attacks was missing for one male and one female HAE patient

In the HAE group, self-reported stress (84.62%) was the most frequent trigger of attacks, followed by physical activity (61.54%) and physical trauma (53.85%). At the time of the study, most (61.54%) were treated with lanadelumab, and one patient was taking berotralstat (71.69%) as a long-term treatment (Table 1). The age at diagnosis was lower in HAE (*M* = 19.1 years, *SD* = 12.6) than in ITP (*M* = 36.5; *SD* = 19.7; *t* (24) = -2.60; *p* = 0.02). In HAE, the average number of attacks over the last six months was 5.27 (*SD* = 5.23, minimum = 0, maximum = 14). In the ITP group, patients reported a mean number of disease relapses of 2.23 (*SD* = 2.71). Compared with ITP, HAE group patients reported a greater mean number of attacks (relapses) over the last six months (*U* = 30.5; *p* = 0.02).

### Reactivity task

In the HAE group, the mean number of peaks in the reactivity task (*M* = 0.92; *SD* = 0.28) was not significantly different from that of ITP (*M* = 0.85; *SD* = 0.38; *U* = 78.0; *p* = 0.58) (Table 2). There was no significant correlation between the reaction times and peak spread of the male patients with HAE (*r* = -0.79, *p* = 0.06) or ITP (*r* = 0.81, *p* = 0.05). The correlation was comparable between the two patient groups (*Z* = -0.05; *p* = 0.96) (Table 2).

**Table 2:** Between group comparison of the experimental task and the effect of sex for HAE

	HAE				ITP			HAE vs. ITP Comparison
	**Global**	Sex		Comparison males vs. females	Global	Sex		
		Female	Male			Female	Male	
**Reactivity task**								
Peaks by period (*M*,* SD*,* n*)	0.92 (0.28) *n* = 13	0.86 (0.38) *n* = 7	1 (0.0) *n* = 6	*U* = 18.0 *p* = 0.44	0.85 (0.38) *n* = 13	0.71 (0.49) *n* = 7	1.0 (0.0) *n* = 6	*U* = 78.0 *p* = 0.58
Correlation of RTs and peak spread	0.42 *p* = 0.18 *n* = 13	0.22 *p* = 0.67 *n* = 7	-0.79 *p* = 0.06 *n* = 6	*z* = 1,72 *p* = 0.09	0.44 *p* = 0.21 *n* = 13	-0.51 *p* = 0.49 *n* = 7	0.81 *p* = 0.05 *n* = 6	*z*= -0,05 *p* = 0.96
**Breathing task**								
HF HRV (*M*,* SD*,* n*) ^a^	835 (920) *n*^a^= 7	545 (517) *n*^a^ = 4	1223 (1322) *n*^a^ = 3	*t* = 0.96 *p* = 0.38	748 (947) *n*^a^ = 9	971 (1121.1) *n*^a^ = 6	303 (32.4) *n*^a^ = 3	*U* = 29.0 *p* = 0.84
Percentage increase breathing base 1 (HF HRV)	1.78% (2.01) *n*^a^ = 7	2.25 (1.79) *n*^a^ *=* 4	1.16 (2.51) *n*^a^ =3	*U* = 3.00 *p* = 0.40	0.51% (1.67) *n*^a^ = 9	-0.08 (0.57) *n*^a^ = 6	1.70 (2.68) *n*^a^ = 3	*U* = 20.0 *p* = 0.25
**Cognitive load task**								
Peaks by period (*M*,* SD*,* n*)	2.77 (1.36) *n* = 13	3.29 (1.50) *n* = 7	2.17 (0.98) *n* = 6	*t* = 1.56 *p* = 0.15	2.77 (1.42) *n* = 13	2.86 (1.86) *n* = 7	2.67 (0.82) *n* = 6	*t*(24) = 0.00 *p* = 1
HF HRV (*M*,* SD*,* n*) ^a^	1712 (2845) *n*^a^ =6	1623 (3172) *n*^a^ = 5	- *n* ^ab^	**-** *n* ^ab^	600 (304) *n*^a^ = 7	672 (312) *n*^a^ = 5	420 (271) *n*^a^ = 2	*U =* 17.0 *p* = 0.63
Percentage increase cognitive base 1 (HF HRV)	9.62% (9.62) *n*^a^ = 6	11.63% (26.1) *n*^a^ = 5	- *n* ^ab^	**-** *n* ^ab^	0.55% (1.79) *n*^a^ = 7	0.91 (2.05) *n*^a^ = 5	-0.36 (0.31) *n*^a^ = 2	*U* = 21 *p* = 1
**Consultation**								
Peaks by period (*M*,* SD*,* n*)	10.6 (5.12) *n* = 13	10.7 (5.02) *n* = 7	10.5 (5.72) *n* = 6	*t*=-0.07 *p* = 0.94	14.4 (7.75) *n* = 13	14.9 (9.19) *n* = 7	13.8 (6.49) *n* = 6	*t*(24)= -1.46 *p* = 0.16
Correlation between physician-perceived stress and peaks by period ^b^	0.43 *p* = 0.14 *n* = 13	0.73 *p* = 0.06 *n* = 7	0.13 *p* = 0.81 *n* = 6	*z* = 1,04 *p* = 0.30	0.03 *p* = 0.93 *n* = 13	-0.03 *p* = 0.95 *n* = 7	-	*z* = 0,99 *p* = 0.32

Note: Abbreviations: N, number of subjects; M, mean; SD, standard deviation; HAE, hereditary angioedema; ITP, immunological thrombocypenia; HF-HRV, high-frequency heart rate variability; RTs, reaction times

Percentage increase cogni-base 1 (HF-HRV): allows determination of whether there is a reduction in HF-HRV during the cognitive phase compared to the baseline

Percentage increase breathing base 1: allows determination of whether there is an increase in HF-HRV during the breathing phase compared to baseline

^a^ for HF HRV, data were missing due to a technical problem with the sensor

^b^ in the cognitive task, due to data loss only one male HAE patient had complete data, hence SD and the comparison of males vs. females were not conducted

^c^ for correlation between physician-perceived stress and peaks by period in the consultation for IT male patients: because all males were evaluated as “0” stress by the doctor; correlation was not calculated

Note: Abbreviations: N, number of subjects; M, mean; SD, standard deviation; HAE, hereditary angioedema; ITP, immunological thrombocypenia; HF-HRV, high-frequency heart rate variability; RTs, reaction times

Percentage increase cogni-base 1 (HF-HRV): allows determination of whether there is a reduction in HF-HRV during the cognitive phase compared to the baseline

### Breathing task

The mean HF-HRV (in ms2) of the HAE group (*M* = 835, *SD* = 920) was comparable to that of the ITP group (*M* = 748, *SD* = 947; *U* = 29.0; *p* = 0.84 (Table 2). The increase in HF-HRV from baseline was 1.78% (*M* = 1.78; *SD* = 2.01) for HAE and 0.51% for ITP (*M* = 0.515, *SD* = 1.67). These values were not significantly different (*U* = 20.0, *p* = 0.25) (Table 2).

### Cognitive load task

The mean number of peaks per period in the cognitive load task was comparable for both groups (HAE (*M* = 2.77, *SD* = 1.36) and ITP (*M* = 2.77; *SD* = 1.42) (*t* (24) = 0.00; *p* = 1.0)) (Table 2). The mean HF-HRV was *M* = 1712 (*SD* = 2845) in the HAE group and *M* = 600 (*SD* = 304) in the ITP group, with no significant difference (*U* = 17.0; *p* = 0.63). Percent increases relative to baseline were also not significantly different between HAE (9.62%) and ITP (0.55%) (*U* = 21.00; *p* = 1) (Table 2).

### The effect of the sex of HAE patients on EDA and HF-HRV in the various tasks

#### Reactivity task

The number of peaks per period did not differ between male (*M* = 1, *SD* = 0.00) and female (*M* = 0.86, *SD* = 0.38; *U* = 18.0; *p* = 0.44) patients with HAE (Table 2).

#### Breathing task

The effect of sex on HF-HRV was not significant (*t* (5) = 0.96; *p* = 0.382) in the breathing task (Table 2). Data were available for only three male patients.

#### Cognitive load task

Due to a malfunction of the photoplethysmography sensor, the cardiac data of several male participants were not available (only one male participant could be measured). We therefore decided not to present the HRV indicators because they were unreliable.

#### Consultation

The number of peaks per period was comparable between HAE (*M* = 10.6; *SD* = 5.12) and ITP (*M* = 14.4; *SD* = 7.75; *t* (24) = -1.46; *p* = 0.16).

In HAE, there was a non-significant positive correlation between patient stress as perceived by thephysician and the number of peaks (objective stress in the patient), but only in female patients (*r* = 0.73; *p* = 0.06). This correlation was not significant in the female patients with ITP (*r* = -0.03; *p* = 0.95). The correlation coefficients for the two populations showed no significant difference (*Z* = 0.99; *p* = 0.32) (Table 2).

### Effect of treatment for HAE (kallikrein inhibitors versus other) on EDA and HF-HRV during the tasks

#### Reactivity task

As shown in Table 3, the number of peaks per period during the reactivity task did not significantly differ between patients receiving kallikrein inhibitors (lanadelumab, berotralsat; *M* = 0.89; *SD* = 0.33) and patients without this treatment (*M* = 1.0; (*SD* not calculable because only one patient was without kallikrein inhibitors), (*U* = 16.0; *p* = 0.62). The correlation between RT and peak spread was similar in patients on kallikrein inhibitors (*r* = 0.30), compared with patients without (*r* = 0.81) (*Z* = -0.76, *p* = 0.45, (Table 3)).

**Table 3:** Comparison of patients with HAE under Kallikrein inhibitors vs. others

	Treatment		
	Under kallikrein inhibitors	Without kallikrein inhibitors	Comparison with vs. without kallikrein inhibitors
**Reactivity task**			
Peaks by period (*M*,* SD*,* n*)	0.89 (0.33) *n* = 9	1.0 (0.00) *n* = 4	*U* = 16.0 *p* = 0.62
Correlation of RTs and peak spread (*r*)	0.3 *p* = 0.51 *n* = 9	0.81 *p* = 0.10 *n* = 4	*z*= -0.76 *p* = 0.45
**Breathing task**			
HF HRV (*M*,* SD*,* n*)	555 (505) *n*^*a*^ = 4	1209 (1339) *n*^*a*^ = 3	*t* = 0.92 *p* = 0.40
**Cognitive load task**			
Peaks by period (*M*,* SD*,* n*)	3.11 (1.36) *n* = 9	2.00 (1.15) *n* = 4	*t* (11) = 1.41 *p* = 0.19
HF HRV (*M*,* SD*,* n*)	2021 (3515) *n*^*a*^ = 4	1093 (1505) *n*^*a*^ = 2	*U* = 3.00 *p* = 0.80

Note. Eight patients were receiving lanadelumab at the time of inclusion, one patient was receiving berotralstat (*N* = 9 patients under kallikrein inhibitors, four patients without) but numbers vary according to measures

^a^. for HF HRV, data were missing due to a technical problem with the sensor

Abbreviations: N, number of subjects; M, mean; SD, standard deviation; HF-HRV, high frequency-heart rate variability; RTs, reaction times

#### Breathing task

HF-HRV (ms2) during the breathing task did not differ significantly between patientson kallikrein inhibitors (*M* = 555; *SD* = 505) and patients without this treatment (*M* = 1209; *SD* = 1339; *t* (5) = 0.92; *p* = 0.40) (Table 3).

#### Cognitive load task

The number of peaks per period of patients with kallikrein inhibitors (*M* = 3.11; *SD* = 1.36) was comparable to that of patients without this treatment (*M* = 2.0; *SD* = 1.15; *t* (11) = 1.41; *p* = 0.19) (Table 3). The same was true for the HF-HRV (*M* = 2021; *SD* = 3515 of patients on kallikrein inhibitors, and *M* = 1093 and *SD* = 1505 in patients without this treatment; *U* = 3.00; *p* = 0.8) (Table 3).

## Discussion

In this exploratory study, we investigated whether patients with HAE were more likely than control patients with ITP to experience dysregulation in the balance between the two branches of the ANS in an experimental task during which HRV and EDA were measured. In contrast to the results of Wu et al. [34], which showed different physiological activation patterns between patients with HAE and healthy controls in a postural stress task, our results did not find significant differences between them in reactivity, cognitive load, or breathing tasks where psychological activation was performed. Our study participants were comparable to those in the Wu et al. [34] study concerning age, but not for the sex distribution or the medical treatments received at the time of the study. Indeed, there were more women in our sample, and more patients were taking kallikrein inhibitors. The differences in our results may thus reflect sex and medical treatment effects.

Our study presents limitations that may explain the non-significant results. The low sample size in this study was primarily due to the rarity of both diseases. In the case of HAE, there are currently 1,500 patients in France. The sample size reflects the real recruitment rate in a study on rare diseases done in a single national reference center. The study uses an innovative, field-based methodology in terms of measuring emotional activity by a wearable device that collects HRV and EDA. Published studies have reported patient numbers of the same order of magnitude [e.g., 44–46]. As such, the results presented in our work should be perceived as exploratory, aiming to inform larger, more targeted studies performed on a multicentric population.

The fact that measurements were carried out in a hospital may have created a physiological state of uncontrollable stress (e.g., fear of arriving late for the medical consultation). There was also considerable variability in the duration of kallikrein inhibitor treatment among the patients with HAE. Because kallikrein inhibitor drugs act on ANS regulation [47–50], treatment duration could have influenced symptoms and quality of life, which, in turn, would affect the ability to manage the disease. Mechanistically, excessive bradykinin generated via the plasma kallikrein–kinin cascade can influence central and peripheral autonomic control: kinins act within the nervous system and have been reported to modulate parasympathetic tone via B2 receptors in brainstem autonomic nuclei, while also engaging nociceptive and vasoregulatory pathways that heighten sympathetic drive during inflammatory states. Thus, by preventing kallikrein-driven bradykinin surges, prophylactic kallikrein inhibition could blunt aberrant autonomic inputs triggered by edema, pain, and threat appraisal [48, 50, 51]. Because emotional stress is also a frequent trigger of attacks, interrupting upstream kallikrein activity plausibly reduces both physiologic (kinin-mediated) and psychophysiologic (anticipatory anxiety) drivers of dysautonomia, which in turn can translate into better quality of life [34]. Although direct trials with ANS endpoints are still limited, randomized and long-term data show that lanadelumab markedly improves patient-reported quality of life and reduces anxiety/depression burden [20]; the hypothesis that psychosocial gains are a credible consequence of restoring autonomic homeostasis through attack prevention and reduced kinin signaling is appealing.

A major limitation of the study is the near-complete loss of cardiac data for the HAE male group during the cognitive load task due to sensor malfunction. Collecting the data in a real-life situation in order to be as close as possible to the patients’ experiences is both a strength and a limitation of our study, as it increases the likelihood that such a data loss would occur. As a consequence, due to such a high rate of data loss in a specific subgroup we cannot conclude that a “sex effect” was present. In the same vein, a trend towards a correlation between physician-perceived stress and physiological measures only in female patients with HAE is interesting, but given the very small sample size we cannot rule out the possibility that true differences were missed due to a lack of statistical power.

While bearing these limitations in mind, and with caution in interpretation, our results suggests some findings. We found a trend toward a linear correlation between the response times and peak spread of male patients. For a population without disease, peak spread is wider when response times are longer, giving rise to a negative correlation [52]. Male patients with HAE showed a negative correlation, as did a population without disease, whereas male patients with ITP showed a positive correlation. Finding a specific pattern of physiological response by patients with ITP was unexpected and may suggest that these patients also experience a high burden of disease. Little data is available on the emotional impact of ITP; however, previous studies have reported anxiety, depression, and post-traumatic stress [53, 54]. By using ITP as a comparison group, we hypothesized that patients with HAE would be more anxious (based on the existing literature), but this may not have been the case.

Regarding the breathing task, the percent increase in HF-HRV compared to the baseline level did not increase in the HAE or ITP groups. This is in contrast to subjects without disease, where an increase in HF-HRV during a breathing task indicates activation of the parasympathetic system [29]. This result may reflect the impact of chronic diseases.

We also assessed whether a physician could correctly perceive the stress experienced by patients during a consultation. Some studies have found alexithymia in patients with HAE [55]. Our results show a trend towards a positive correlation, not reaching significance, between the perceived stress (by the physician) and the number of peaks found in the patient during the consultation phase, but only in the female patients with HAE. Although caution should be applied when interpreting this result due to the small sample size, it suggests that when female patientsexperience stress (a physiological measure), they express it through verbal and non-verbal behaviors in such a way that aphysician can adequately perceive it [56]. Male patients may use more suppressive expression strategies, hindering an appropriate assessment of their the emotional state [57].

The absence of significant results between the HAE and ITP groups suggests that patients with ITP may also have emotional regulation disorders. Although ITP attacks are usually not emotionally-triggered, patients remain burdened with living with a chronic, attack-prone and potentially fatal rare condition, which may have biased our results and neutralized significant differences between the disease groups, hindering the observation of pathophysiologically specific alterations in emotional regulation. As such, it would be interesting to account for this potential bias by including a “healthy control” group or by comparing HAE and ITP with and without anxiety-depressive disorders to determine whether our findings are linked to deregulation of the ANS or to the involvement of the anxiety-depressive state. In order to collect enough data to have sufficient statistical power, conducting the study in several centers would be crucial. Combining measures of stress via physiological biomarkers (HRV, EDA) with psychological measures of emotion and its regulation would also be interesting, as well as improving the experimental setup. The data losses we encountered stemmed from issues related to the use of the device (improperly positioned wristband, depleted battery) and from connection problems that could not be resolved in time. The data collection software, which transmits information either through a mobile phone or a direct computer connection, requires access to a secure remote server. In addition, part of the data collection took place during the pandemic and immediate post-pandemic periods when experimental conditions were particularly challenging. More broadly, conducting experiments in a hospital setting presents significant difficulties. It initially seemed advantageous to take advantage of scheduled hospital visits to conduct the study. However, these conditions are far from ideal for the participants: patients are often anxious prior to meeting with their physician, and it was precisely at that moment that we carried out the behavioral measurements. In light of this experience, it appears preferable for future studies to be conducted in a more welcoming and comfortable environment that is clearly separated from the hospital context and medical consultation in order to reduce anxiety-related or context-driven biases. This would lead to a better understanding of the role of the ANS in the emotional regulation of patients with HAE.

This study is part of a larger research project that is focused on the emotion regulation of patients with HAE, and will be complemented by qualitative (interviews) and quantitative (questionnaires) data.

## Supplementary Information

Below is the link to the electronic supplementary material.

Supplementary Material 1

## Data Availability

The datasets generated and/or analyzed during the current study are not publicly available to protect study participant privacy. They are available from the corresponding author on reasonable request.

## Abbreviations

HAE: Hereditary angioedema
ITP: Immune thrombocytopenia
EDA: Electrodermalactivity
HRV: Heart rate variability
