# Supplementary material for: Physiological evaluation of the emotional regulation of patients with hereditary angioedema
Source: Biopsychosoc Med. 2026 Jan 6;20:1. doi: 10.1186/s13030-025-00348-6 (PMC12777275; doi:10.1186/s13030-025-00348-6)
Supplement: Supplementary file 1 — Supplementary Material 1 [file 13030_2025_348_MOESM1_ESM.docx]

Supplementary Material 1. Task descriptions and indicators

| **Task** | **Time (in seconds)** |
| --- | --- |
| **Baseline Level**  Completion of the sociodemographic data sheet | 120 |
| *Recovery* | 60 |
| **Reactivity Task**  Pressing a key as soon as a stimulus appears on the screen: to measure sympathetic activity  Indicators: number of peaks per period (phasic EDA) | 15 |
| *Recovery* | 60 |
| **Breathing Task**  Short inspiration and long expiration breathing exercise to activate the parasympathetic system  Indicators HF-HRV (ms2) during the breathing task | 120 |
| *Recovery* | 60 |
| **Cognitive Task**  A mathematical operation is displayed on the screen and the participant gives the result in writing  Indicators: number of peaks (phasic EDA) per period and HF-HRV (ms2) during the cognitive load task | 120 |
| *Recovery* | 60 |

Note. EDA = Electrodermal Activity

HF HRV = High-Frequency Heart Rate Variability
